# Editorial: Advanced analytic techniques in developmental neuroscience

**DOI:** 10.3389/fnins.2022.1070337

**Published:** 2023-01-05

**Authors:** Jacob Levman, Emi Takahashi

**Affiliations:** ^1^Department of Computer Science, St. Francis Xavier University, Antigonish, NS, Canada; ^2^Athinoula A. Martinos Center for Biomedical Imaging, Massachusetts General Hospital and Harvard Medical School, Charlestown, MA, United States; ^3^Research, Innovation and Discovery Center for Clinical Research, Nova Scotia Health Authority, Halifax, NS, Canada; ^4^Department of Radiology, Harvard Medical School, Boston, MA, United States

**Keywords:** analytics, machine learning, pathologies, development, neuroscience

Modern developmental neuroscience research is fundamentally multidisciplinary and includes the extensive use of advanced analytic techniques. These analyses typically employ computationally demanding methods, inclusive of advanced techniques, such as machine learning, combined with animal models as well as imaging of human patient populations, to further our understanding of brain development. In this special topic, we have published inter-related manuscripts focused on a number of important developmental conditions, each involving the use of advanced analytic techniques. Machine learning is emerging as a powerful technique for analyzing neurological data and our collection has included a manuscript on the application of machine learning to neurological magnetic resonance imaging (MRI) examinations in a schizophrenia population (Levman et al.), inclusive of public domain software to assist researchers in applying machine learning to their analyses. Additionally, a neural network model was developed to reveal motoric effects associated with exposure to nicotine in an approach for movement disorder diagnoses (Torabi et al.). Advanced methods for quantitatively analyzing brain development are also available, including automated software for extracting biomarkers of potential interest. An advanced computational analytic technique known as connectomics, which maps major fiber tracts across the brain, has been used to identify a potential association between hyperconnectivity and symptom severity in autism (Ouyang et al.). A review article covering the quantitative analysis of Rett syndrome was included (Shiohama and Tsujimura), and an additional study focused on using advanced tools in support of a comprehensive volumetric analysis of a mouse model of Rett syndrome was also included in our article collection (Akaba et al.). Finally, an analysis was performed using perfusion contrast for spatial normalization of arterial spin labeling MRI examinations in a pediatric craniosynostosis population (de Planque et al.).

The use of advanced analytic techniques has the potential to assist in improving our understanding of neurological development. For example, the analysis of a schizophrenia population (Levman et al.) revealed correlations between patient depression and the thickness of the right medial orbitofrontal cortex, and the machine learning analysis reported on a variety of brain regions with potentially abnormal development. The application of machine learning in studying nicotine exposure (Torabi et al.) has resulted in new technology capable of identifying movement alterations in posture, movement initiation and repeated movements. The application of advanced analytics in autism (Ouyang et al.) has reported that patient symptom severity is correlated with aberrant hyperconnectivity. Advanced analysis of a mouse model of Rett syndrome has revealed hemispheric asymmetry in several brain regions (Akaba et al.), and a related review article on imaging in Rett syndrome provides an overview of advanced methods being actively used to characterize neurological data in this domain (Shiohama and Tsujimura). Finally, the perfusion contrast study demonstrates the potential for the use of advanced analytic techniques for the characterization of patients with pediatric craniosynostosis (de Planque et al.).

In summary, this Research Topic demonstrates the use of advanced analytical techniques in a wide range of fields. The techniques used in each of the studies presented here are not limited to the field of that particular paper, but can be used in a wide range of applications. As analytic techniques for studying the brain continue to improve in terms of image acquisition, post processing analyses, animal models and machine learning, we expect our ability to evaluate and monitor brain development to continue to improve.

## Author contributions

ET proposed the collection and revised and approved the editorial. ET and JL both administered the article collection. JL authored the editorial. All authors contributed to the article and approved the submitted version.

